# Association between Unintentional Interpersonal Postural Coordination Produced by Interpersonal Light Touch and the Intensity of Social Relationship

**DOI:** 10.3389/fpsyg.2017.01993

**Published:** 2017-11-23

**Authors:** Tomoya Ishigaki, Ryota Imai, Shu Morioka

**Affiliations:** ^1^Department of Neurorehabilitation, Graduate School of Health Sciences, Kio University, Nara, Japan; ^2^Department of Home-visit Rehabilitation, Fit-care Home-visit Nursing Station, Osaka, Japan; ^3^Department of Rehabilitation, Higashiikoma Hospital, Nara, Japan; ^4^Neuro Rehabilitation Research Center, Kio University, Nara, Japan

**Keywords:** postural sway, touch, interpersonal postural coordination, social relationship, closeness, rapport

## Abstract

Interpersonal postural coordination (IPC) produced by interpersonal light touch (ILT), whereby time-series variations in the postural sway between two people unintentionally resemble each other, may be a possible social interaction. From a sociopsychological standpoint, close mutual behavioral coordination is recognized as “social glue,” which represents the closeness of relationships and contributes to the building of a good rapport. Therefore, we hypothesized that if IPC functions as social glue, then IPC produced by ILT also represents a social relationship. Participants were dyadic pairs with a preexisting social relationship (acquaintance, friend, or best-friend), and we assessed the closeness between the partners. Postural sway in two quiet standing conditions—no touch (NT) and ILT (a mutual light touch with <1 N) condition—was concurrently measured with the side-by-side standing position, and the association of IPC with intradyadic closeness (rapport) was analyzed using hierarchical linear modeling. The results showed that unintentional IPC was higher in both axes of the ILT condition than in NT condition. Additionally, IPC in the mediolateral axis (the partner side) of the ILT condition was positively correlated with intradyadic closeness, whereas that in the anteroposterior axis (the non-partner side) showed a negative association. As expected, IPC represented intradyadic closeness (rapport). Results indicate that, in unintentional IPC produced by ILT, the priority of processing sensory feedback for postural control, which is received from the individual and a partner, is modulated depending on the rapport in interactional coupled feedback loops between the two individuals (i.e., good rapport increases the degree of taking in feedback from a partner). Thus, unintentional IPC produced by ILT functions as social glue, and it provides an understanding of the sociopsychological aspect in the human-to-human postural coordination mechanism.

## Introduction

Sensory information from multiple systems is necessary for adapting postural orientation to a dynamically changing environment (Horak, 2006). In particular, touching a fixed object using a part of the body with slight force (<1 N), which provides haptic information regarding body movement and spatial orientation in relation to the object, reduces postural sway without mechanical support (Holden et al., 1994; Jeka, 1997; Kouzaki and Masani, 2008). This reduction is observed when not only the object being touched is fixed material but also it is a spontaneously moving human. Johannsen et al. (2009) first reported that interpersonal light touch (ILT) in quiet standing reduces postural sway and causes interpersonal postural coordination (IPC) with slight force, by which the time-series variation between the postural sway of two people unintentionally resembling each other. Furthermore, when an object with sinusoidal oscillations or with the prerecorded postural sway of another person is played back on a touching device, the postural sway of a subject synchronizes with the oscillations of the object (Wing et al., 2011). In other words, postural sway of the subject is oriented to an external reference point depending on the feedback information that a person receives from the touched objects. Consequently, there is a decrease or increase in the sway depending on the touching situation. The mechanisms of altering postural sway and IPC produced by ILT have been demonstrated by model simulation using interactional coupled feedback loops (Reynolds and Osler, 2014). Regarding how the postural sway is influenced, regardless of partner sway it is possible that a reduced sway occurs with only a light finger touch, because a relative reference point is obtained in most cases of ILT with a quiet standing posture (Reynolds and Osler, 2014). Specifically, both these situations cause decreased sway in the subject—the situation of ILT in which a subject takes a quiet bipedal standing posture and a partner takes an unstable tandem standing posture (Johannsen et al., 2012), and a subject takes open eyes and a partner takes an unstable closed eyes standing posture (Reynolds and Osler, 2014). On the other hand, IPC produced by ILT is achieved by the subject receiving the partner’s sway information as a feedback for the subject’s own postural control and by orienting their postural sway to one another on the basis of the interactional feedback (Reynolds and Osler, 2014).

Such unintentional IPC in quiet standing has also been reported as occurring via sensory information other than haptic. Visual information through eye contact in the face-to-face position produces IPC (Okazaki et al., 2015). In addition, verbal interaction during puzzle solving produces IPC related to partner sway, regardless of visual feedback regarding partner sway; in other words, IPC might be produced by sharing patterns of conversation (Shockley et al., 2003, 2007). These previous behavioral interaction studies suggest that IPC is functionally and unintentionally mediated by visual, auditory, or haptic information. Although these studies have revealed a kinematic aspect, other essential aspects of human-to-human interaction in IPC have not been demonstrated. Thus, we propose the possibility of a sociopsychological aspect.

When we communicate, we may unconsciously demonstrate close behavioral coordination, such as mimicry and synchrony in good social relationships; and this in turn contributes to the building of a good rapport without our being aware of this (Lakin et al., 2003; Chartrand and Lakin, 2013). Therefore, behavioral coordination is referred to as “social glue” because it is two-sided (Lakin et al., 2003). Furthermore, much evidence exists that interpersonal touch itself is a strong means of non-verbal communication (e.g., in modulating the tendency to comply with requests, in affecting people’s attitudes toward specific services, in creating bonds between couples or groups, and in strengthening romantic relationships) (Gallace and Spence, 2010). Therefore, considering the social function of behavioral coordination and interpersonal touch, we hypothesize that, if IPC, which is produced by ILT, functions as social glue, then it can also represent aspects of social relationships, such as rapport. Although several studies (Miles et al., 2010; Zhao et al., 2015; Brambilla et al., 2016) have reported that social relationships established artificially before an interaction task can influence the degree of interpersonal motor coordination between the individuals involved, it has not yet been clarified what influence social relationships may have on unintentional IPC produced by ILT. Investigating this association would aid further understanding of the mechanism of IPC mediated by haptic information.

To examine our hypothesis mentioned above, we use the ILT paradigm to avoid mechanical link (forced touch) and focus on a sensory link (Reynolds and Osler, 2014). In addition, to capture IPC without intentional cooperation, we employed quiet standing as an implicit motor task because non-stationary postural sway in quiet standing is spontaneously and unconsciously generated (Carroll and Freedman, 1993). Moreover, we took this approach to avoid creating bias to an explicit and dynamic IPC task through ILT. The following fact exemplifies our approach—when performing a cooperative task that keeps rhythmic sway stable through ILT produced by hearing auditory cues in pair (i.e., an explicit and dynamic task), a skilled dance pair has a superior ability to keep rhythmic sway more stable than a non-dancer pair (Sofianidis et al., 2012, 2015). Moreover, when performing the same task in a mixed pair (i.e., skilled dancer and non-dancer), the skilled dancer leads the non-dancer through sway (Sofianidis et al., 2014). These findings indicate that the difference in individual motor ability in a dynamic IPC task might create bias in determining the association between sociopsychological factors and IPC produced by ILT. In addition, regarding the impact of sociopsychological factors on behavioral coordination, previous studies have used artificially controlled experimental approaches with an unknown partner or virtual agent, such as one of the partners intentionally being late for the experiment or arriving on time (Miles et al., 2010), the virtual agent being attractive (or unattractive) (Zhao et al., 2015), or depicting the partner as honest (versus dishonest) or friendly (versus unfriendly) (Brambilla et al., 2016). However, in social relationships, rapport is essentially a natural bond in daily living; therefore, targeting preexisting natural relationships might be more suitable than using artificially controlled experimental approaches. Thus, the purpose of the current study was to determine whether unintentional IPC produced by ILT in quiet standing is associated with rapport in preexisting natural social relationships.

## Materials and Methods

### Participants

Twenty-four dyads consisting of 48 healthy students (mean age 21.3 ± SD 3.1 years) participated in this study. When recruiting participant dyads, we set the inclusion criteria as follows: individuals within the dyad knew each other as an acquaintance, friend, or best-friend (mean duration of knowledge 28.3 ± 15.6 months) before the experiment, and the dyads consisted of the same sex (11 male dyads and 13 female dyads). Further, through self-reporting the number of each type of social relationship was controlled to a feasible extent (five male dyads out of nine acquaintance dyads, four male dyads out of nine friend dyads, two male dyads out of six best-friend dyads) to include a wide range of relationships. There was no significant difference between the sex and type of social relationships (*P* = 0.48, Pearson’s Chi-square test). All participants provided informed written consent. This study conformed to the Declaration of Helsinki and was approved by the Ethical Committee of Kio University (approval number: H28-35).

### Experimental Procedure

The participant dyads entered an anteroom together, and we briefly explained the experimental procedure to minimize bias. This deliberate explanation was conducted to avoid intentional IPC; therefore, participants were not informed about the experimental purpose or the phenomenon of IPC produced by ILT. Following the explanation, the dyads were separated to prevent any communication, and they answered psychological questionnaires regarding their closeness with their partner to assess rapport. The Inclusion of Other in the Self (IOS) scale (Aron et al., 1992), the love-liking scale (Rubin, 1970; Fujiwara et al., 1983), and the Friendship function scale (*in Japanese*) (Tanno, 2008) were used to assess closeness in this study. The IOS scale, which is easy for respondents to understand and is the most reliable measure for assessing subjective closeness (Gächter et al., 2015), asks respondents to assess their relationship with a specific individual by selecting one out of seven pairs of increasingly overlapping circles (i.e., a wider overlapping circle mean greater closeness) (Aron et al., 1992). The love-liking scale has two subscales (the love scale evaluates affection and the liking scale evaluates positive feeling), and each subscale consists of 13 questions (Rubin, 1970; Fujiwara et al., 1983). Answers are on a 9-point scale, from 1 = “not at all true” to 9 = “definitely true.” Total scores can therefore range between 13 and 117 for each scale. The Friendship function scale was developed in Japanese and has nine subscales (“relaxation, comfort,” “entertainment,” “prospect of continuing relationship,” “emotional bonds,” “consultation, self-disclosure,” “support,” “affirmation, acceptance,” “learning, self-development,” and “importance in life”), and each subscale consists of five questions (Tanno, 2008). Answers are on a 5-point scale, from 1 = “not at all true” to 5 = “true.” The total score summed with the average score of each subscale is used for analysis; in other words, the total score can be between 5 and 25.

The participant dyads were then moved into the same experimental room, and their postural sway was concurrently measured. After the measurement, the participants were separated again, and they answered a questionnaire on whether they recognized that their own postural sway had coordinated with their partner’s.

**FIGURE 1:**
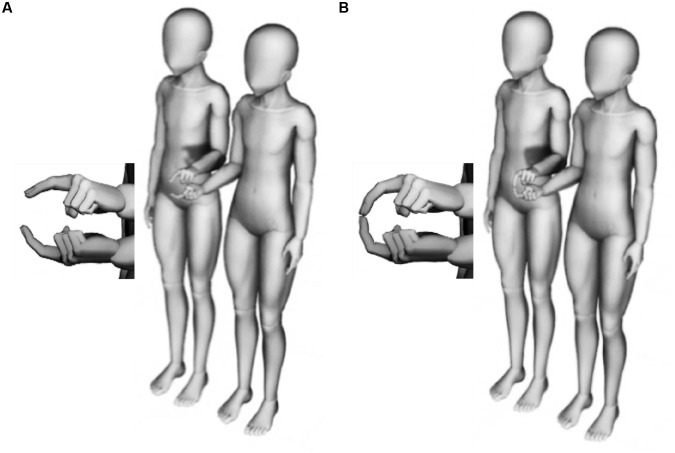
Standing conditions. Participants stood on two independent platforms, positioned side-by-side, with a space of 10 cm between the participants at the shoulder, facing in a forward direction, with eyes closed, the elbow of the partner side flexed at around 90° with the index finger extended, and the arm on the other side hanging down. The side of the taller participant was set randomly. The hand of the taller participant on the partner side was positioned above the shorter participant’s fingertip in a pronation position, and that of the shorter participant was positioned below the taller one in a supine position. The participants were instructed to stand in a relaxed manner, and postural sway was measured in the following two standing conditions: (1) the no touch (NT) condition, in which the participants kept a narrow-base standing posture in a normal bipedal position with a 5 cm interheel interval **(A)** and (2) the ILT condition, in addition to the NT condition, in which participants mutually touched their index fingertips (<1 N) **(B)**. The human model drawn in **Figure 1** was made by using “DesignDoll,” which may be freely used for commercial or non-commercial purposes (http://terawell.net/terawell/).

**FIGURE 2:**
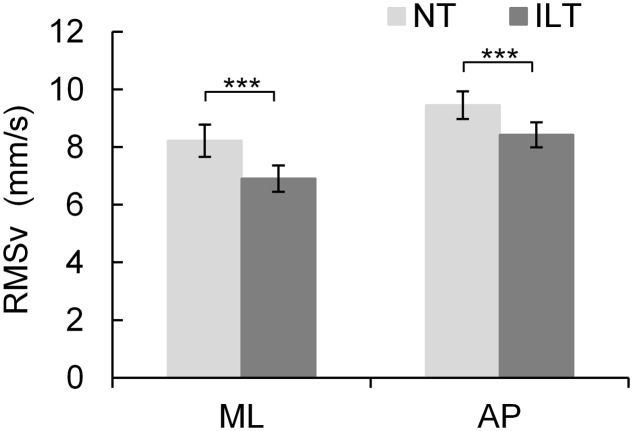
Average RMSv (mm/s) (± standard error) of all participants (*n* = 48, individual). NT (*light gray*), no touch condition; ILT (*dark gray*), interpersonal light touch condition. ^∗∗∗^ RMSv in both axes (ML and AP) of the ILT condition was significantly lower than that of NT (*P* < 0.001). RMSv, root mean square of center of foot pressure velocity; ML, mediolateral; AP, anteroposterior.

For the measurement of postural sway, the participant dyads stood on two independent platforms, positioned side-by-side, with a space of 10 cm between the participants at the shoulder, facing in a forward direction, with eyes closed, the elbow on the partner’s side flexed at around 90° with the index finger extended, and the arm on the other side hanging down. The side on which the taller participant stands was set on left or right sides in a random order. The hand of the taller participant on the partner’s side was positioned above the shorter participant’s fingertip in a pronation position, and that of the shorter participant was positioned below the taller one in a supine position. The participants were instructed to stand in a relaxed manner, and postural sway was measured in the following two standing conditions: (1) the no touch (NT) condition, in which participants kept a narrow-base standing posture in a normal bipedal position with a 5 cm interheel interval (**Figure 1A**) and (2) the ILT condition, in addition to the NT condition, in which the participants mutually touched their index fingertips (<1 N) (**Figure 1B**). The above conditions were similar to the part of previous studies (i.e., no-touch or ILT in normal bipedal standing) to allow for the comparison of the results (Johannsen et al., 2009, 2012). The NT condition was first performed for 30 s followed by the ILT condition for the same duration. Three trials were conducted in the same order for each condition.

A dual stabilometer platform (Twin Gravicorder G-6100; Anima Co., Ltd., Tokyo, Japan) was used to record the center of foot pressure (CoP) displacement (sampling 100 Hz). To measure the touch force between touching fingertips in Newtons, a sheet-type force sensor (Flexi Force B201; Tekscan Inc., South Boston, MA, United States) was attached to the fingertip of the taller participant. Data recording of touch force (sampling 100 Hz) and device synchronization were controlled using a digital analog converter (USB-6009; National Instruments Corp., Austin, TX, United States) operated with LabVIEW 2013 (National Instruments Corp., Austin, TX, United States).

### Behavioral Data Analysis

All data were analyzed using MATLAB R2014a (The MathWorks Ins., Natick, MA, United States). Time-series data of touch force were low-pass filtered (5 Hz, fourth-order, zero-phase shift, Butterworth). Similarly, CoP displacement data were low-pass filtered (10 Hz, fourth-order, zero-phase shift, Butterworth) and differentiated to generate CoP velocity (CoPv) for rendering the displacement data as stationary. To assess postural sway, the root mean square value of CoPv (RMSv) was calculated. Further, cross-correlation analysis was performed with the “coeff” option being activated as the cross-covariance function (XCOV in MATLAB) so as to assess the IPC of each standing condition from the CoPv time-series trace in each axis (mediolateral: coronal plane; anteroposterior: sagittal plane). The “coeff” option was activated for normalizing the sequence so that the autocorrelation at zero lag equal 1. The resulting cross-correlation coefficients vary between 1 and -1, and these values represent the full coordination as in-phase (positive) and anti-phase (negative), respectively. The peak cross-correlation coefficient (Xcorr value) and corresponding time lag (range, ±1,000 ms; +, taller precede; -, shorter precede) were extracted. All analyzed variables were averaged in each condition and axis. The analyses for CoP were similar to previous studies to compare results (Johannsen et al., 2009, 2012; Reynolds and Osler, 2014).

### Statistical Analysis

Multipsychological questionnaires assessing the social relationship toward the partner were used for assessing the relationship comprehensively in this study. Characteristics of these scales resemble each other and practically are showing moderate correlation with each other (**Table 1**, Spearman’s rank correlation coefficient, *n* = 48, individual). Therefore, to validly determine the association between social relationships and IPC, it is necessary to avoid type I errors (repetitive statistical test) and analyze the common components of variance in each scale. For these reasons, principal component analysis (PCA) was conducted for dimension reduction and to extract the principal component score of the main component as the “closeness value.” In this analysis, the grand total score summed with the love-liking scale (Love-Liking score) was used as the entered variable in addition to other variables (IOS scale and Friendship function scale), because these two variables were moderately correlated with each other (**Table 1**).

**Table 1:** Spearman’s rank correlation coefficient matrix of psychological questionnaires (*n* = 48).

Scale	1	2	3	4
1: IOS		0.44^∗∗∗^	0.26^†^	0.75^∗∗∗^
2: Love			0.63^∗∗∗^	0.64^∗∗∗^
3: Liking				0.46^∗∗∗^
4: Friendship function				


*IOS, Inclusion of Other in the Self. ^∗∗∗^*P* < 0.001; ^†^*P* < 0.10.*

To compare the RMSv (*n* = 48, individual), the Xcorr value (*n* = 24, dyad), and the time lag (*n* = 24, dyad) between standing conditions in each axis, a paired *t*-test was used. To test whether the time lag differed from 0 lag, a one-sample (0 lag) *t*-test (*n* = 24, dyad) was used. Statistical analyses were conducted using IBM SPSS Statistics for Windows ver. 23 (IBM Corp., Armonk, NY, United States). *P*-values of less than 0.05 were considered statistically significant. Data is presented as mean ± SD.

### Hierarchical Linear Modeling

When analyzing dyadic data (e.g., individuals nested within a dyad), a hierarchical linear modeling (HLM) approach is appropriate (Raudenbush and Bryk, 2002). The closeness value data sampled in this study has a within-dyad (Level 1) and between-dyad (Level 2) hierarchical structure, which can show the interdependence of within-dyad trends. This tendency might have occurred owing to the recruitment of participant dyads with a preexisting social relationship in this study. Therefore, to examine the association between IPC produced by ILT and intradyadic closeness (i.e., rapport), control of the interdependence of within-dyad closeness is required. HLM can control the interdependence of within-dyad closeness and analyze between-dyad variance.

In this study, HLM 7 (Scientific Software International Inc., Skokie, IL, United States) was used with the restricted maximum likelihood estimation method. In the model design, regarding the explanatory variable, only the fixed effect was modeled to examine the hypothesis; hence, two models were analyzed. The first model was the “Null model,” whereby only the objective variable (closeness value) and individual and dyad identification data were entered. The aim of the Null model was to calculate the intraclass correlation (ICC), which is the mean rate of within-dyad interdependence with respect to all variance. The formula is as follows: ICC = (variance of intercept)/(variance of residual + variance of intercept). When the ICC value is greater than or equal to 0.1, the validity criteria for using HLM are met (Raudenbush and Bryk, 2002). Next, the “Hypothesis model” was analyzed to test whether IPC produced by ILT was associated with intradyadic closeness. In this model, the Level 1 explanatory variable (age) and Level 2 explanatory variables (sex, duration of knowledge, and Xcorr value of the ILT condition in both axes) were entered as the fixed effect. Additionally, in accordance with the recommendation of the analytical method (Kreft et al., 1995), the Level 1 explanatory variable was processed into group-mean centered variable and the Level 2 explanatory variables were processed into grand-mean centered variables except for sex.

## Results

### Psychological Questionnaires and Closeness Value

As a result of PCA, the first component showed a large proportion (76.1%) and a significant eigenvalue = 2.28. Moreover, every entered variable showed principal component loading not less than 0.8 (**Table 2**). Conversely, the other components (second and third) showed a low proportion and a non-significant eigenvalue (<1.0) (**Table 2**). Thus, only the 1st component was employed and we extracted the principal component score as the “closeness value.” The descriptive statistics values of the psychological questionnaires and the closeness value are shown in **Table 3**.

**Table 2:** Principal component analysis results (*n* = 48).

	Principal component loading		
Scale			
	First component	Second component	Third component
Friendship function	0.94	0.07	-0.32
IOS	0.86	0.46	0.23
Love-liking	0.81	-0.57	0.14

Eigenvalue	2.28	0.54	0.18
Proportion (%)	76.10	18.08	5.82

*IOS, Inclusion of Other in the Self.*

**Table 3:** Descriptive statistics values of psychological questionnaires and closeness value.

	Group			
Scale				Total (*n* = 48)
	Acquaintances (*n* = 18)	Friends (*n* = 18)	Best-friends (*n* = 12)
IOS (point)	3.06 (1.21) [-5]	3.78 (1.00) [2-6]	5.08 (0.79) [4-7]	3.83 (1.29) [1-7]
Love (point)	67.89 (16.48) [37-99]	66.50 (16.47) [39-99]	80.33 (23.17) [46-115]	70.48 (18.85) [37-115]
Liking (point)	74.11 (11.55) [47-93]	65.61 (16.42) [35-104]	82.33 (21.22) [44-112]	72.98 (17.17) [35-112]
Love-liking (point)	142 (26.61) [84-192]	132.11 (28.79) [77-183]	162.67 (43.08) [90-226]	143.46 (33.68) [77-226]
Friendship function (point)	15.80 (3.45) [8.11-20.89]	18.01 (2.40) [14.33-22.22]	21.31 (2.27) [18.33-24.67]	18.00 (3.50) [8.11-24.67]
Closeness value	-0.50 (0.95) [-2.62-1.14]	-0.13 (0.67) [-0.95-1.55]	0.96 (0.86) [-0.07-2.46]	0.00 (1.00) [-2.62-2.46]

*IOS, Inclusion of Other in the Self. Mean (±SD) [Min–Max].*

### Touch Force

The touch forces of all trials in the ILT condition were less than 1 N, and the mean was 0.31 ± 0.14 (min–max: 0.16–0.61) N.

### Postural Sway

The RMSv of the ILT condition was significantly lower than that of the NT condition in the mediolateral (ML) axis (NT: 8.22 ± SD 0.56 mm/s, ILT: 6.91 ± 0.46 mm/s; *P* < 0.001; **Figure 2**) and in the anteroposterior (AP) axis (NT: 9.46 ± 0.48 mm/s, LT: 8.43 ± 0.43 mm/s; *P* < 0.001; **Figure 2**). Overall, touch reduced postural sway by 12.28% (ML: 14.15 ± 14.32%; AP: 10.41 ± 11.25%).

### Cross-Correlation for IPC

**FIGURE 3:**
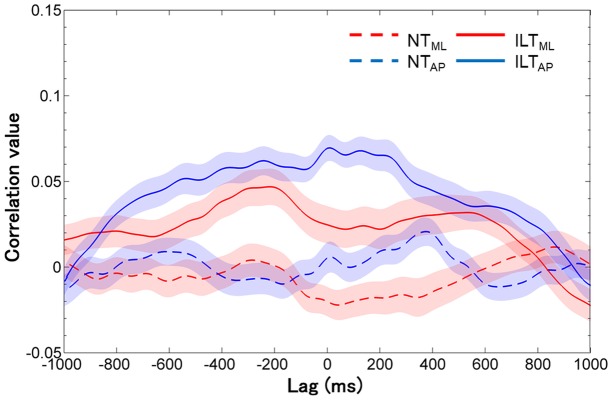
Grand-averaged cross-correlation curve data of all trials in each axis and condition (band, ± standard error). Cross-correlation coefficients of the ILT condition (*solid line*) in both axes (ML: *red*, AP: *blue*) were higher than that of the NT condition (*dotted line*) throughout the time lag, and the peak cross-correlation coefficients were approximately ±300 ms in both axes of the ILT condition. NT, no touch; ILT, interpersonal light touch; ML, mediolateral; AP, anteroposterior; correlation value, cross-correlation coefficient; lag, time lag.

**FIGURE 4:**
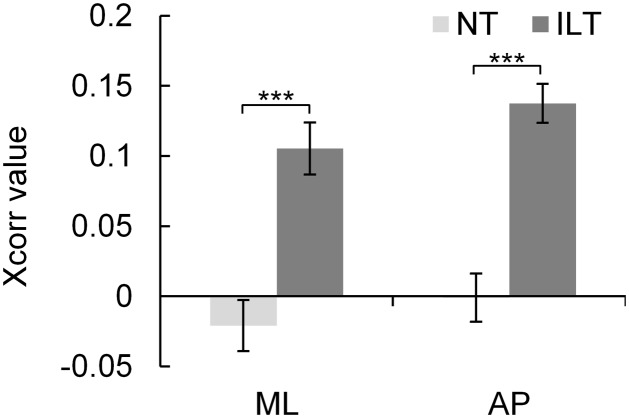
Average Xcorr value (± standard error) of all dyads (*n* = 24, dyad). ^∗∗∗^ Xcorr values in both axes (ML and AP) of the ILT condition (*dark gray*) were significantly higher than that of NT (*light gray*) (*P* < 0.001). NT, no touch; ILT, interpersonal light touch; ML, mediolateral; AP, anteroposterior; Xcorr value, peak cross-correlation coefficient.

The grand-averaged cross-correlation curve data of all trials in each axis and condition are presented in **Figure 3**. The results indicate that cross-correlation coefficients of the ILT condition in both axes were higher than that of the NT condition throughout the time lag. The Xcorr value peaked at around ±300 ms in both axes of the ILT condition, which was significantly higher than that of the NT condition in the ML axis (NT: mean -0.02 ± 0.09; ILT: 0.11 ± 0.09; *P* < 0.001; **Figure 4**) and in the AP axis (NT: 0.00 ± 0.09; ILT: 0.14 ± 0.07; *P* < 0.001; **Figure 4**). Regarding the time lag corresponding to the Xcorr value, none of the axes showed a significant difference from 0 lag (ML axis: NT, 104.31 ± 386.82 ms, ILT, -16.94 ± 344.61 ms; AP axis: NT, -37.22 ± 326.21 ms, ILT, 107.08 ± 323.75 ms; all *P* > 0.1).

### Questionnaire for IPC

None of the participants noticed whether their postural sway was coordinated with that of their partner in the ILT condition.

### Hierarchical Linear Modeling

**FIGURE 5:**
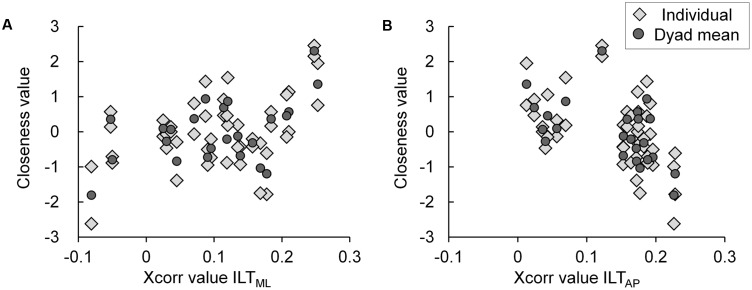
Scatter plots showing the data distribution between the Xcorr value of the ILT condition and the closeness value. In the individual (*light gray diamond*) and dyad mean (*dark gray circle*) distribution plots, **(A)** a positive association between the Xcorr value and the closeness value in the ML axis and **(B)** a negative association in the AP axis are shown. ILT, interpersonal light touch; ML, mediolateral; AP, anteroposterior; Xcorr value, peak cross-correlation coefficient.

The data distribution between the Xcorr values of the ILT condition and the closeness values is presented in **Figures 5A,B**. Although the features of variance between individual and dyad means are not completely matched, the tendency of both distribution plots are similar (**Figures 5A,B**). In other words, in both the individual and the dyad mean plot distributions, a positive association between the Xcorr value and the closeness value was found in the ML axis (**Figure 5A**), whereas a negative association was seen in the AP axis (**Figure 5B**).

The results of the Null model are presented in **Table 4A** and indicate that the data in this study has a high within-dyad interdependence (ICC = 0.61). The Hypothesis model is presented in **Table 4B** and indicates that the Level 1 explanatory variable of age did not significantly correlate with the Level 1 closeness value (*P* > 0.10). In contrast, the Xcorr value of the ML axis in the ILT condition was significantly positively associated with the Level 2 closeness value (*P* < 0.05) (**Table 4B**). Moreover, the Xcorr value of the AP axis in the ILT condition was significantly negatively associated with the Level 2 closeness value (*P* < 0.05) (**Table 4B**).

**Table 4:** Hierarchical linear modeling results of closeness value (*n* = 48 individual; *n* = 24 dyad).

(A) Null model					(B) Hypothesis model				
Random effect					Random effect				
	*SD*	Variance	*X^2^*	*P*		*SD*	Variance	*X^2^*	*P*
Intercept (*df* = 23) Residual	0.78	0.62	94.37	<0.001	Intercept (*df* = 19) Residual	0.54	0.29	45.90	<0.001
	0.63	0.40				0.64	0.41		
	
**Fixed effect**					**Fixed effect**				
	**β**	***SE***	***t***	***P***		**β**	***SE***	***t***	***P***

Intercept Level 1 (*df* = 23)	0.00	0.18	0.00	1.00	Intercept Level 1 (*df* = 23)	0.09	0.22	0.44	0.67
									
					Age	-0.01	0.05	-0.22	0.83
					Level 2 (*df* = 19)				
					Sex (male = 0, female = 1)	-0.18	0.30	-0.58	0.57
					Duration of knowledge	0.01	0.01	1.42	0.17
					Xcorr value ILT_ML_	4.61	1.70	2.71	0.01
					Xcorr value ILT_AP_	-5.40	2.25	-2.40	0.03
	
ICC = 0.61, Deviance = 124.47					ICC = 0.41, Deviance = 117.17				

*SD, standard deviation; SE, standard error; β, unstandardized coefficient; ICC, intraclass correlation; Xcorr value, peak cross-correlation coefficient; ILT, interpersonal light touch; ML, mediolateral; AP, anteroposterior.*

## Discussion

In this study, the main component of variance in the multipsychological questionnaires assessing social relationships was able to extract “the closeness value” appropriately owing to conducting the PCA (**Table 2**). Moreover, the recruited participant dyads already naturally had a wide range of preexisting social relationships, which were generally ranked in a positive order according to the scores of the psychological questionnaires and the closeness value (i.e., best-friend dyads had the highest score, and acquaintance or friend dyads had the lowest) (**Table 3**). Therefore, the sampled data was considered valid for analyzing the hypothesized association because a wide variance of closeness in the between-dyad could be inferred. Furthermore, although the features of variance between the individual and the dyad mean were not completely matched, the association trends were similar (**Figure 5**), and the Null model in the HLM analysis showed a high ICC of 0.61 (**Table 4A**). Hence, the results indicated a high interdependence of intradyadic closeness and methodological validity, which are required for HLM analysis (Raudenbush and Bryk, 2002). Besides, in the ILT condition, we considered it as successfully capturing the influence of sensory feedback on postural control because the touch forces were less than 1N in all trials (Holden et al., 1994; Kouzaki and Masani, 2008; Reynolds and Osler, 2014). Moreover, this sensory feedback led IPC to result in a higher Xcorr value in the ILT condition than NT condition (**Figure 4**). Certainly, the difference of the Xcorr values between ILT and NT conditions are clear; however, the Xcorr values of ILT conditions are low in both axes (approximately 0.1–0.15). Regarding this concern, we considered that one of the reasons for these results is due to the use of CoPv data for cross-correlation analysis. Whereas using the differentiated data (CoPv) for the analysis improves the data stationarity; low frequency signals with large amplitude and consisting of the main component of the CoP displacement is attenuated and the minute fluctuation per data sampling is increased. Hence, it becomes more difficult to extract the characteristic of correlation than the case of using the CoP displacement. Additionally, since cross-correlation analysis in this study was not focusing on the dynamics of IPC changing on the time domain but on the characteristic of IPC shown throughout the trial, the extracted Xcorr values represent the generalized value roughly. For these reasons, we considered that low Xcorr values were shown in this study. Nevertheless, the Xcorr values were also 0.1 approximately in the ILT condition of the previous study that employed the same data analysis with this study (Reynolds and Osler, 2014). Further, considering the Xcorr values were shown only 0.2 approximately even under the condition that both parties grasp the shoulder and shoulder, and generate IPC by mechanical coupling in the previous study (Reynolds and Osler, 2014), we will be able to estimate that the intensity of IPC in the ILT condition was about half of the mechanical coupling, and this indicates that adequate and moderate IPC was generated. Consequently, we considered that the Xcorr values in this study appropriately reflected the characteristic of IPC in the trial even though the values were low. In addition, none of the participants recognized that their postural sway indicated IPC in the post-experiment questionnaire. In other words, IPC by ILT was induced unintentionally. Hence, we believe that those results are reliable for supporting the hypothesis of this study.

The clear difference between this study and previous ones is the aspect of the recruited participants. Participant dyads in this study were paired according to preexisting social relationships, whereas that was not the case in previous studies (in which participants were not described [Johannsen et al., 2009] or were only acquaintances [Johannsen et al., 2012; Reynolds and Osler, 2014]). Regarding the RMSv of the ILT condition, it was lower than that of the NT condition in both axes, and the reduction rate was approximately 12% (10–14%). This rate in quiet standing is similar to that of previous studies at approximately 13–18% (Johannsen et al., 2009, 2012; Reynolds and Osler, 2014). Therefore, regardless of the participants feature, we concluded that the sway reduction observed in this study arises from the same mechanism demonstrated previously, namely, being able to obtain a relative reference point by ILT (Reynolds and Osler, 2014).

**FIGURE 6:**
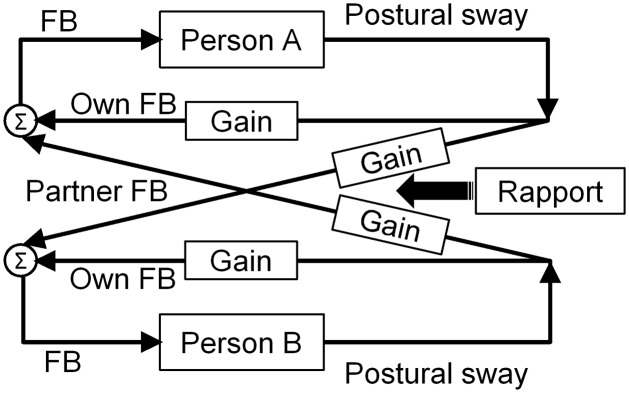
Model of interactional coupled feedback loops simplified for explaining the influence of rapport. This is a modification of the model by Reynolds and Osler (2014) to show the results of this study. Basically, each participant has an independent feedback loop, namely, own feedback (Own FB). However, when participants perform interpersonal light touch, interactional coupled feedback loops are connected to each other, namely, partner feedback (Partner FB). Each feedback loop equips the gain functions to control the degree of receiving information. These gain functions are gain for Own FB and gain for Partner FB. Moreover, these gains have a trade-off function (e.g., increase gain for sensory feedback from the partner when there is decreased gain for one’s own feedback). Rapport directly controls gain for Partner FB, and then gain for Own FB is indirectly controlled depending on the trade-off rule.

Concerning the influence of the difference in the participants feature on the Xcorr value representing IPC, a previous study only showed a higher Xcorr value in the AP axis of the ILT condition; however, in this study the values were higher in both axes (Johannsen et al., 2012; Reynolds and Osler, 2014). **Figure 5** shows that the Xcorr values of the ILT condition are dispersed in the positive range corresponding to the closeness value (individual and dyad mean), and these showed positive associations in the ML axis and negative associations in the AP axis. In the Hypothesis model, which controlled for within-dyad interdependence and analyzed between-dyad variance, the Xcorr value in the ML axis of the ILT condition was positively associated with intradyadic closeness, and, conversely, that of the AP axis showed a negative association (**Table 4B**). Taking into account the difference in the participants feature, and assuming that IPC represents social relationships, high Xcorr values might have been shown in both axes as overall averages. Furthermore, previous studies have demonstrated that prior relationships before an experimental task influence behavioral coordination and suggest that social relationship modulates the degree of receiving the partner’s information in terms of behavioral coordination (Miles et al., 2010; Zhao et al., 2015; Brambilla et al., 2016). Considering this relevant finding, we consider that social relationships might function as a “gain controller” for modulating the degree of sensory information processing of the individual’s and the partner’s behavior control. Such a process has been demonstrated by using model simulations when IPC is produced by ILT and has a trade-off function that increases the gain in sensory feedback from the partner when there is decreased gain in the individual’s own feedback (Reynolds and Osler, 2014). Considering this system, we believe that intradyadic closeness functions as a gain controller for modulating partner feedback; that is, good rapport increases the gain toward partner feedback and decreases the individual’s own feedback. Additionally, there are tendencies to mutually modulate gain similarly because the sampled closeness had high within-dyad interdependence. Therefore, we conclude that the association of IPC with intradyadic closeness acts as “social glue” in this study. The simplified model based on Reynolds and Osler (2014) is presented in **Figure 6** for the purpose of explaining the influence of rapport.

Furthermore, as to the inverse relations depending on the axis, IPC might be induced according to the standing position in which the partners interact. Nearer interpersonal distance is known to correlate with better friendship and attraction (Sundstrom and Altman, 1976), suggesting spatial distance influences social interactions. The design of this study was a side-by-side position with a very close distance that deeply invaded the participants’ personal space in the ML direction (**Figure 1**). For this reason, unlike in previous studies (Johannsen et al., 2012; Reynolds and Osler, 2014), we surmise that the results of this study show a high Xcorr value in the ML axis of the ILT condition and a positive association with intradyadic closeness. Regarding the negative association in the AP axis, it may be a result of preferring the influence in the ML axis; namely, it may be attributed to a secondary effect. However, further research is required for demonstrating this possibility.

Regarding the time lags, the results of the ILT condition in each axis showed no significant difference with 0 lag in the statistical analysis; however, those standard deviations were large and the grand-averaged cross-correlation curves showed an unclear peak at 0 lag (**Figure 3**). Accordingly, although there was a seemingly 0 lag, it was inferred as an averaged result by the positive, negative, and fully synchronized time lags. We assume this inconsistent result arose because the interaction task did not have set roles regarding a leader and a follower. In other words, there is not always a fixed leader role throughout a trial; instead, the leader might dynamically change or fully synchronize. Our being unable to mention the dynamics of IPC is a methodological limitation that is related to the use of cross-correlation analysis. Furthermore, our inability to make a clear and pertinent analysis related to the dynamics of IPC, might show the result of inconsistent time lag and low Xcorr value. A more clear interaction task and precise time-series analysis that focuses on such roles and their time domain is needed in the future study.

## Conclusion

We revealed that unintentional IPC in quiet standing produced by ILT is associated with intradyadic closeness in preexisting social relationships. This finding suggests that rapport functions as a gain controller for modulating feedback from a partner in a model of interactional coupled feedback loops. The present findings provide an understanding of the sociopsychological aspect in a human-to-human postural coordination mechanism. Moreover, such a system is based on an interactional feedback model in IPC, as indicated in previous studies, and it seems that attributing to such a postural coordination mechanism is common in not only haptic feedback (i.e., ILT) but also visual feedback (Okazaki et al., 2015) and dynamic coordination tasks (Gueugnon et al., 2016). Therefore, we propose that it is possible to apply the present findings to other sensory modalities and postural coordination tasks.

## Author Contributions

TI and SM contributed to the development of the study hypothesis. All authors designed the study. TI performed the data analysis and drafted the initial manuscript with input from SM. All authors contributed to data collection and interpretation and critically reviewed the manuscript. All authors approved the final version of the manuscript and agree to be accountable for all aspects of the study in ensuring that questions related to the accuracy or integrity of any part of the study are appropriately investigated and resolved.

## Conflict of Interest Statement

The authors declare that the research was conducted in the absence of any commercial or financial relationships that could be construed as a potential conflict of interest.
